# Phenotypic and genetic characterization of a patient with a de novo interstitial 14q24.1q24.3 deletion

**DOI:** 10.1186/1755-8166-7-49

**Published:** 2014-07-21

**Authors:** Elisa Tassano, Andrea Accogli, Serena Panigada, Patrizia Ronchetto, Cristina Cuoco, Giorgio Gimelli

**Affiliations:** 1 Laboratorio di Citogenetica, Istituto G.Gaslini, L.go G.Gaslini 5, Genoa 16147, Italy; 2 Pediatric Pulmonology and Allergy Unit, Istituto Giannina Gaslini, Genoa, Italy

**Keywords:** Interstitial 14q24.1q24.3 deletion, De novo, Array-CGH, Genotype-phenotype correlation

## Abstract

**Background:**

Interstitial deletions of chromosome bands 14q24.1q24.3 are very rare with only three reported cases.

**Results:**

We describe a 7-year-old boy with a 5.345 Mb *de novo* interstitial deletion at 14q24.1q24.3 band detected by array-CGH who had a complex phenotype characterized by seizures, congenital heart defects, dysmorphisms, psychomotor delay, and bronchopulmonary, skeletal, and brain anomalies.

**Conclusion:**

The deleted region contains numerous genes, but we focused our attention on three of them (C14orf169, *NUMB,* and *PSEN1*), which could account, at least partially, for the phenotype of the boy. We therefore discuss the involvement of these genes and the observed phenotype compared to that of previously described patients.

## Background

Interstitial deletions involving chromosome band 14q24.1q24.3 are very rare. The use of array-CGH in routine cytogenetic diagnostics allowed the detection of pathogenic copy number variants (CNVs), which contribute to the delineation of new genomic disorders. However, there are chromosome regions in which no well-characterized aberrations were found, such as 14q24.1q24.3. Recently, Oehl-Jaschkowitz et al., [1] described the first three unrelated patients with overlapping *de novo* interstitial 14q23.1q24.3 deletion characterized by array-CGH. All three patients had mild intellectual disability, congenital heart defect, brachydactyly, hypertelorism, broad nasal bridge and thin upper lip.

Here, we report on a *de novo* 14q24.1q24.3 deletion in a boy with complex phenotype characterized by seizures, congenital heart defects, dysmorphisms, psychomotor delay, and bronchopulmonary, skeletal, and brain anomalies. We compare the phenotype of our patient with that of previously reported patients and discuss the role of the deleted genes in order to investigate the possibility of a genotype-phenotype correlation.

## Case presentation

**Figure 1:**
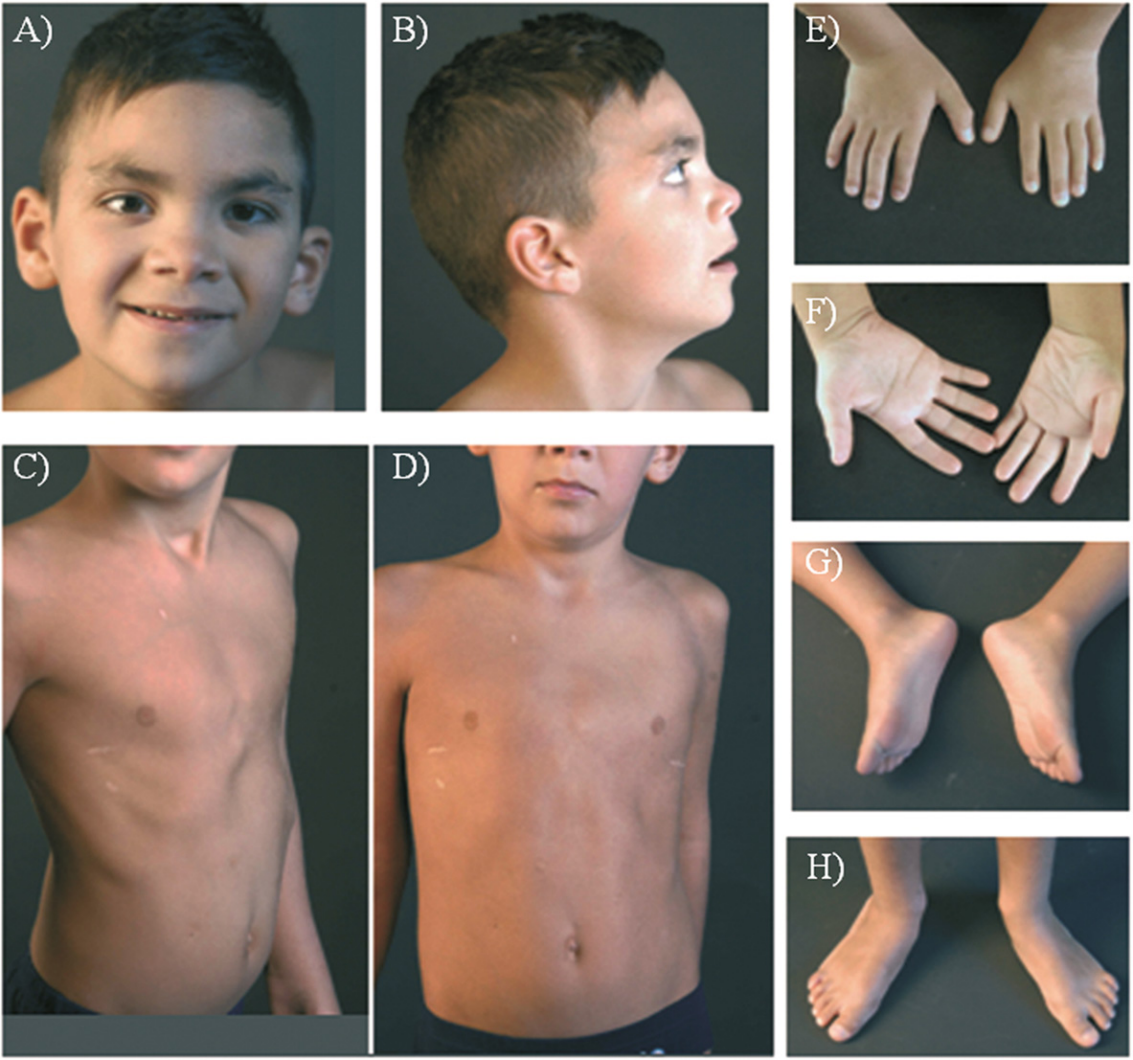
**Patient at the age of 7 years. A) B)** Note broad and sparse eyebrows, convergent strabismus, broad nasal bridge and hypertelorism,nose with broad columella and nares, ears with prominent helix and antihelix and large lobes, long philtrum, thin upper lip, short neck, low set posterior hairline; **C) D)** Pectus excavatum; **E) F)** Short fingers and nails, broad thumbs; **G) H)** Short toes and nails, broad big toes and flat feet.

**Figure 2:**
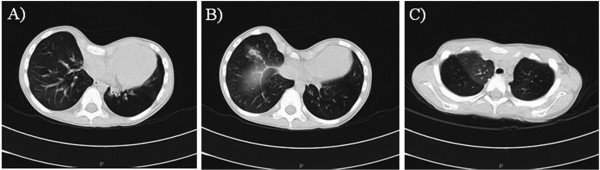
**Chest CT scan before thoracic surgery. A) B)** severe pectus excavatum with a Haller index of 4.07, bronchiectases and ground glass opacities **C)** area of consolidation and air trapping.

The proband is a 7-year-old Italian male born to non consanguineous, healthy parents with another older healthy child. The child was born at 37 weeks of gestation by caesarean section after an uneventful pregnancy. Birth weight was 3050 g (90th centile), length 54 cm (90th centile), and head circumference (OFC) 36 cm (50th centile). Hypotonia, poor feeding, and mild dysmorphisms were observed from the first days of life. He was referred to the Istituto Giannina Gaslini at one month of life because of bronchiolitis and pneumonia complicated by respiratory arrest treated with intubation, ventilatory support, and oxygen supplementation for 2 months. Chest CT scans showed areas of parenchymal consolidation caused by ventilation defects characterized by thick mucous secretions in the upper right lobe and hilar areas bilaterally. During hospitalisation, the patient had seizures documented by abnormal areas in temporal lobes on EEG and was started on phenobarbital. Metabolic screening, abdominal ultrasound, and routine blood biochemical tests were normal. Cardiac ultrasound showed atrial septal defect with spontaneous closure at follow-up. Fundus oculi and visually evoked potentials (VEP) were normal. Because of gastroesophageal reflux and antral gastritis, PPI therapy was started and Nissen fundoplication was necessary at the age of three years. During the first years of life, the child experienced recurrent episodes of wheezing and upper and lower respiratory tract infections complicated by respiratory insufficiency with desaturation requiring admission to emergency care. Sweat test and genetic testing for cystic fibrosis were normal. Serum immunoglobulin levels showed transient childhood hypogammaglobulinemia. Lymphocyte counts, lymphocyte proliferation test, tests for surfactant deficiency, and laryngotracheobronchoscopy with bronchoalveolar lavage (BAL) were normal. Nasal brushing showed ciliary dyskinesia due to chronic inflammation. Periodic chest CT scans showed bronchial wall thickening, bronchiectasis, areas of consolidation with ground glass opacities and air trapping especially in the medium and upper right lobes. Due to recurrent pulmonary infections, PEP-mask (positive expiratory pressure) physiotherapy, and treatment with bronchodilators, inhaled corticosteroids, and anti-leukotriene agents were started. Physical examination during the first years of life showed broad and sparse eyebrows, convergent strabismus, broad nasal bridge and hypertelorism, nose with broad columella and nares, ears with prominent helix and antihelix and large lobes, long philtrum, thin upper lip, short neck, low set posterior hairline. Skeletal evaluation showed pectus excavatum, short fingers and toes, broad thumbs and big toes, short nails, joint hyperlaxity of lower limbs, and flat feet (Figure 1). Moreover, neurological examination showed mild psychomotor delay and especially speech delay, attention deficit, and hyperactivity. Since these features were associated with focal epilepsy, initially poorly responsive to conventional drugs, brain MRI was performed at the age of four years, showing mild symmetrical enlargement of supratentorial ventricular cavities and enlargement of fronto-insular periencephalic spaces (Figure 2). At the age of six years thoracic surgery for pectus excavatum was performed with ensuing improvement of respiratory dynamics and reduction in the frequency of respiratory infections.

## Results

**Figure 3:**
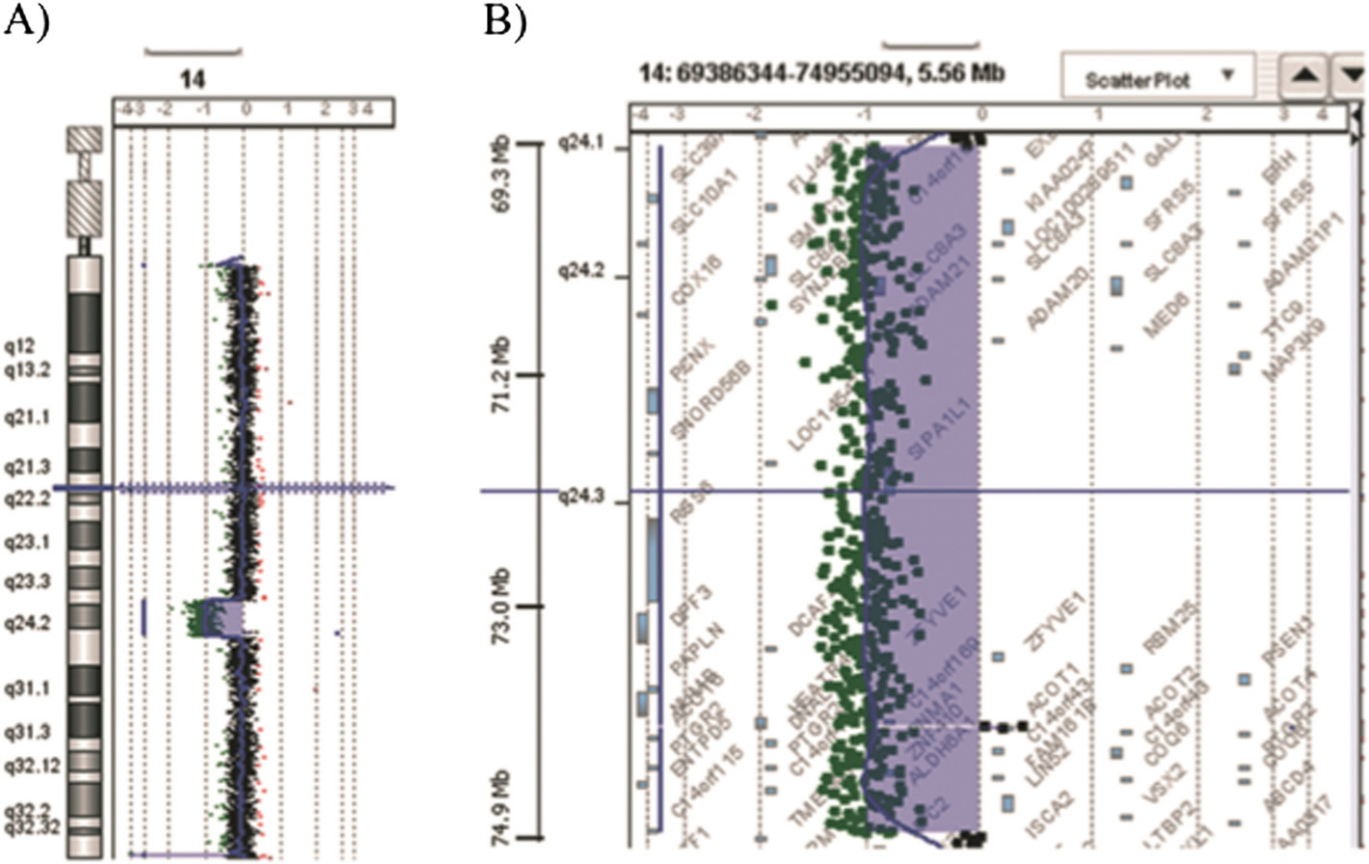
**Results of array-CGH analysis.** Array CGH revealed a de novo interstitial deletion of 5.345 Mb at q24.1q24.3 band of a chromosome 14: arr[hg19]14q24.1q24.3(69,500,246-74,845,355)x1. **A)** Array-CGH profile of patient's chromosome 14 showing the 14q deletion, **B)** enlargement of the deleted region 14q.

Cytogenetic analysis, performed on GTG-banded metaphases from cultured lymphocytes of the patient and his parents, showed normal karyotypes. Considering the phenotypic abnormalities of the patient, array CGH analysis was performed, showing a 5.345 Mb *de novo* interstitial deletion at 14q24.1q24.3 bands. The deletion spanned from probe A_16_P40228390 (69,500,246 bps) to probe A_16_P20096718 (74,845,355 bps) flanked by probe A_16_P02937773 (69,482,103 bps) and probe A_16_P20096719 (74,852,834 bps) (Figure 3). The complete region covered by these deletions harbours more than 50 RefSeq genes with both known and unknown functions, but we focused our attention on the genes C14orf169 (NO66) (nucleolar protein 66; MIM 611919), *NUMB* (numb Drosophila homolog; MIM 603728), and *PSEN1* (presenilin 1; MIM 104311) which could be candidate genes for the patient’s phenotype in addition to those discussed by Oehl-Jaschkowitz et al., 2014 [1].

## Discussion

**Figure 4:**
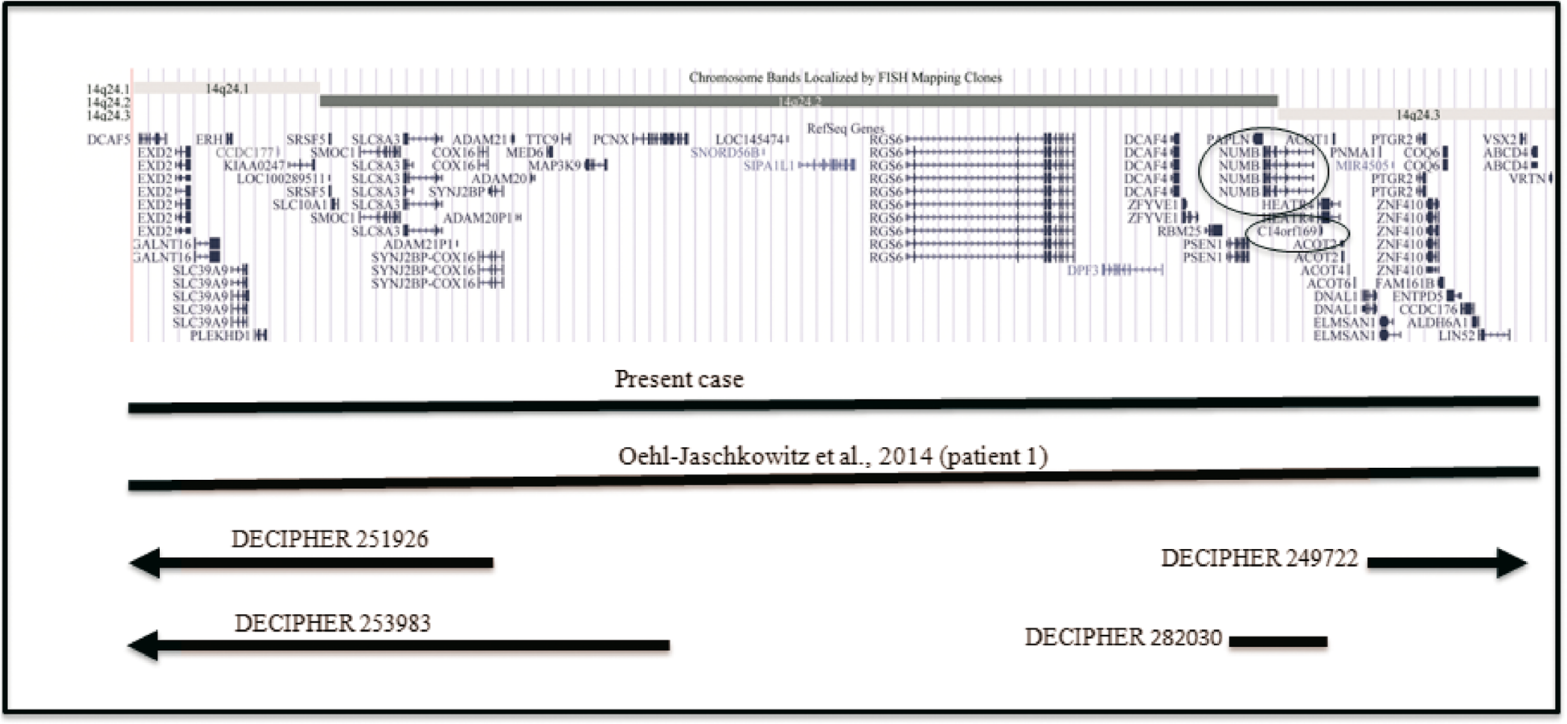
**Overview of the 14q24.1q24.3 region and its gene contents, according to the UCSC Genome Browser (GRCh37/hg19 assembly).** The circles indicate the genes, which could be responsible for the phenotypic features of the 14q24.1q24.3 deletion patients. Bars indicate the deleted region of patients.

Interstitial deletions of chromosome 14 involving the 14q24.2 band are very rare, and most deletions reported in the literature involve the proximal or distal end of chromosome 14. To our knowledge, Oehl-Jaschkowitz et al. [1] reported the first three patients, but only patient 1 had a deletion overlapping that of our case, while patients 2 (DECIPHER 251926) and 3 (DECIPHER 250043) showed a smaller deletion (Figure 4). Clinical features of the four patients are reported in Table 1. The two patients with overlapping deletion (our patient and patient 1) presented both atrial septal defect and wheezing or asthma, short fingers, hypertelorism, thin upper lip, language delay, and intellectual disability. Similarly to the other two patients, patient 2 and 3 showed different cardiac defects, hypertelorism, language delay, and mild intellectual disability. Another two subjects, with partial deletions, have been reported in DECIPHER database (http://decipher.sanger.ac.uk). The patient DECIPHER 249722 showed a 2.07 Mb deletion at band 14q24.3 (Figure 4) and presented preauricular skin tag, behavioural/psychiatric abnormalities, intellectual disability, and proportionate short stature. Another patient (DECIPHER 282030) presented a 185.2 Kb partial deletion at band 14q24.2 including only two OMIM genes: *PSEN1* and *NUMB* (Figure 4). The only reported clinical features were autism and delayed speech.

**Table 1:** Clinical Features of the patients with 14q24.1q24.3 deletion

**Clinical features**	**Present case**	**Patient 1 Oehl-Jaschkowitz et al., **[1]	**Patient 2 Oehl-Jaschkowitz et al., **[1]	**Patient 3 Oehl-Jaschkowitz et al., **[1]
Position of 14q24 deletion (hg19)	chr14:69,500,246-74,852,834	chr14:69,379,727-74,826,674	chr14: 68,609,319-70,919,016	chr14:68,816,535-71,659,567
Size of 14q24 deletion	5.3 Mb	5.4 Mb	2.3 Mb	2.8 Mb
Sex	Male	Male	Male	Female
Seizures	+	-	-	-
Bronchopulmonary anomalies	Respiratory insufficiency. Bronchial hyperactivity	Asthma	-	-
Congenital heart disease	Atrial septal defect	Atrial septal defect	Pulmonary atresia with a ventricular septal defect, anteriorly-set aorta and severe stenosis of the pulmonary arterial confluence	Trunctus arteriosus
Facial dysmorphisms	Broad and sparse eyebrows, convergent strabismus, broad nasal bridge and hypertelorism, nose with columella and naris broad, ears with prominent helix and antihelix and large lobe, long philtrum, thin upper lip, short neck, low set posterior hairline	Hypertelorism, high nasal bridge, long and flat philtrum, thin upper lip.	Hypertelorism, small nose, thin upper lip, downslanting palpebral fissures.	Hypertelorism, mild synophrys, epicanthic folds and downslanting palpebral fissures, midface hypoplasia, thin lips
Hand and foot anomalies	Short fingers and nails of feet and hands, broad toes and thumbs, valgus -flat feet	Bilateral hypoplastic thumbs, short and tapering fingers and cutaneous syndactyly of the fingers.	Very small hands that were narrow across the metacarpophalangeal joints and proximally-set thumbs. The feet were small with minimal 2-3 toe syndactyly and an over-curved 4^th^ toenail bilaterally.	-
Pychomotor delay	+	-	-	+
Developmental delay	-	+	+	+
Language delay	+	+	+	+
Attention deficiency and hyperactivity	+	NR	NR	NR
Intellectual disability	+	Mild	+	+
Skeletal malformations	Pectus excavatum, joint hyperlaxity of lower limbs, valgus-flat feet	Limited extension and supination of elbows	Short arms, limited elbow extension with bilateral dislocation of the radial heads	Hyperlaxity of the fingers and elbows
Brain abnormality	Mild symmetrical enlargement of supratentorial ventricular cavities and enlargement of fronto-insular periencephalic spaces	NR	NR	NR

NR = not reported.

Given the large number of deleted genes comprised in the deletion, the comparison of our patient with a second case could contribute to the identification of candidate genes responsible for the phenotypic features of patients with 14q24.1q24.3 deletion.

The clinical features shared by the three patients reported by Oehl-Jaschkowitz et al. [1] were congenital heart defects, brachydactyly, mild intellectual disability, and facial dysmorphic signs. These authors suggested a possible causative role of *SMOC1* and *DCAF5* genes in the phenotypic features of their patients.

It is interesting to note that both patients 2 and 3, carrying a smaller deletion, had more severe heart defects and patient 2 had anomalies of hands and feet. This could be due to the haploinsufficiency of more genes or to their different expressivity. However, we can hypothesize that other genes or genetic factors could modify the phenotype in each particular case.

In addition to the above-mentioned genes, we considered *C14orf169* (*NO66*), *NUMB*, and *PSEN1* as possible causative genes for the phenotype of our patient and for patient 1 reported by Oehl-Jaschkowitz et al. [1].

The gene product of *NO66* (*C14orf169*) is a jumonji C-containing protein identified as Osterix-interacting polypeptides expressed in bone that exhibits an *in vitro* demethylase activity with dual specificity for lysines 4 and 36 of histone H3. According to Sinha et al. [2], interactions of NO66 demethylase with Osterix should be considered physiologically significant in regulating osteoblast differentiation through modulation of Osterix activity. The authors concluded that NO66 helps gene repression through histone demethylation and/or formation of a repressor complex, which results in multilayered control of chromatin architecture of specific osteoblast genes [3].

Another interesting gene is *NUMB*, which is a cell fate determinant and plays a key role in asymmetric cell division (ACD) versus symmetric cell division (SCD) [4-6].

In ACD, a dividing mother cell segregates cell fate determinants asymmetrically into only one of the two daughter cells, and this process is indeed crucial for balancing self-renewal, cell differentiation, and correct spatial and temporal specification of cell lineages during development [7-9].

In the lung, ACD plays an essential role in mediating the balance between lung epithelial stem/progenitor cell maintenance and differentiation of cell populations at distal epithelial tips during lung development [10,11].

Lethal defects of gas diffusion capacity such as the common congenital forms of lung hypoplasia and bronchopulmonary dysplasia as well as the limited capacity of the lung to recover from these defects could be explained by a significant deficiency of stem/progenitor cells [11,12]. Therefore, proper balance between self-renewal and differentiation of lung-specific progenitors, which is mediated by ACD, is essential for normal morphogenesis and regeneration of the lung.

Moreover, loss of epithelial cell polarity is also involved in lung epithelial cancers and chronic obstructive pulmonary disease, which are likewise related to disruption of lung epithelial differentiation and cellular function [13].

As regards neurogenesis, it has been reported that Numb and Numblike (Numbl) are functionally related proteins that critically regulate progenitor differentiation and neuroepithelial integrity during embryonic neurogenesis [14-17]. They function during neural precursor ACD to antagonize Notch function in one of the daughter cells [18]. In mouse, the loss of Numb and Numbl causes premature progenitor cell depletion and, consequently, a highly specific malformation of the neocortex and hippocampus [14].

Recently, Zhao et al. [19] studied NUMB functions in cardiac progenitor cell differentiation and cardiac morphogenesis. Heart development is a spatiotemporal multistep morphogenetic process that depends on the addition of progenitor cells from four different sources, including cells from the first heart field and the second heart field (FHF and SHF), cells derived from cardiac neural crest cells, and cells derived from the pro-epicardial organ [20-24].

Perturbations in different cardiac cell populations determine a spectrum of congenital heart defects. The posterior SHF contributes to create chambre septation. Abnormal differentiation and development of the cells of this area were found associated with atrial septal defect and atrioventricular septal defect [25,26]. In fact, as demonstrated in knockout mice, the deletion of *NUMB* and *NUMBL* in SHF-derived cells resulted in atrioventricular septation defects, which indicates their role in cardiac morphogenesis [19].

Finally, another deleted gene in our patient, *PSEN1* (Presenilin 1), is the catalytic component of the γ-secretase complex, a membrane-embedded aspartyl protease that plays a central role in biology and in the pathogenesis of Alzheimer's disease.

Mutations in the *PSEN1* gene are the most common cause of autosomal dominant Alzheimer's disease (AD), with around 180 mutations described to date. *PSEN1* AD has a broad clinical phenotype, encompassing not only dementia but also a variety of other neurological features that may include epileptic seizures [27]. Recently, in a transgenic mouse model, it has been shown that altered expression of Numb isoforms in vulnerable neurons occurs during AD pathogenesis, which suggests a role for Numb in the disease process [28].

The other genes in this interval with known disease associations are *DNAL1*, *COQ6, ALDH6A1, CHX10,* and *ABCD4.* Their mutations cause autosomal recessive syndromes and no abnormalities have been reported in heterozygous carriers.

Therefore, in our opinion *NUMB* and *PSEN1* could be suggestive of cardiac, neurological, and respiratory phenotypes. Moreover, *NO66* deletion could play a role in the skeletal anomalies of our patient.

## Conclusions

In conclusion, we identified a new very rare case of a 14q24.1q24.3 deletion in a boy affected by cardiac, neurological, bronchopulmonary, and skeletal anomalies. This region encompasses about 50 RefSeq genes. We suggest that *NUMB*, *PSEN1*, and *NO66* genes, in addition to those reported by Oehl-Jaschkowitz et al. [1], may play a role in the phenotypic features of our patient. Furthermore, the patient 1 [1] and our patient are the first human cases of a deletion of the *NUMB* gene, which are consistent with its importance for the cardiac, neurologic and lung normal development. On the other hand, we cannot exclude some influence of the many other genes included in the deleted region.

## Methods

Standard GTG banding was performed at a resolution of 400-550 bands on metaphase chromosomes from peripheral blood lymphocytes of the patient and his parents. Molecular karyotyping was performed on the proband and his parents using Human Genome CGH Microarray Kit G3 180 (Agilent Technologies, Palo Alto, USA) with ~13 Kb overall median probe spacing. Labelling and hybridization were performed following the protocols provided by the manufacturers. A graphical overview was obtained using the Agilent Genomic Workbench Lite Edition Software 6.5.0.18.

## Consent

Written informed consent was obtained from the patient’s parents for publication of this paper and any accompanying images. A copy of the written consent is available for review by the Editor-in-Chief of this journal.

## Competing interests

The authors declare that they have no competing interests.

## Authors’ contributions

All authors have made substantial contributions to conception and design, acquisition of data, analysis and interpretation of data. All authors have been involved in drafting the manuscript and revising it critically for important intellectual content. All authors read and approved the final manuscript.
